# REporting recommendations for tumour MARKer prognostic studies (REMARK)

**DOI:** 10.1038/sj.bjc.6602678

**Published:** 2005-08-02

**Authors:** L M McShane, D G Altman, W Sauerbrei, S E Taube, M Gion, G M Clark

**Affiliations:** 1 US National Cancer Institute, Bethesda, MD 20892, USA; 2 Cancer Research UK Medical Statistics Group, Centre for Statistics in Medicine, Wolfson College, Oxford OX2 6UD, UK; 3 Institut fuer Medizinische Biometrie und Medizinische Informatik, Universitaetsklinikum Freiburg, 79104 Freiburg, Germany; 4 Centro Regionale Indicatori Biochimici di Tumoure, Ospedale Civile, 30122 Venezia, Italy; 5 OSI Pharmaceuticals, Inc., Boulder, CO 80301, USA

**Keywords:** tumour marker, guidelines, REMARK, NCI, EORTC, prognostic

## Abstract

Despite years of research and hundreds of reports on tumour markers in oncology, the number of markers that have emerged as clinically useful is pitifully small. Often initially reported studies of a marker show great promise, but subsequent studies on the same or related markers yield inconsistent conclusions or stand in direct contradiction to the promising results. It is imperative that we attempt to understand the reasons that multiple studies of the same marker lead to differing conclusions. A variety of methodological problems have been cited to explain these discrepancies. Unfortunately, many tumour marker studies have not been reported in a rigorous fashion, and published articles often lack sufficient information to allow adequate assessment of the quality of the study or the generalisability of the study results. The development of guidelines for the reporting of tumour marker studies was a major recommendation of the US National Cancer Institute and the European Organisation for Research and Treatment of Cancer (NCI-EORTC) First International Meeting on Cancer Diagnostics in 2000. Similar to the successful CONSORT initiative for randomised trials and the STARD statement for diagnostic studies, we suggest guidelines to provide relevant information about the study design, preplanned hypotheses, patient and specimen characteristics, assay methods, and statistical analysis methods. In addition, the guidelines suggest helpful presentations of data and important elements to include in discussions. The goal of these guidelines is to encourage transparent and complete reporting so that the relevant information will be available to others to help them to judge the usefulness of the data and understand the context in which the conclusions apply.

Despite years of research and hundreds of reports on tumour markers in oncology, the number of markers that have emerged as clinically useful is pitifully small (Hayes *et al*, 1996; Bast *et al*, 2001; Schilsky and Taube, 2002). Often initially reported studies of a marker show great promise, but subsequent studies on the same or related markers yield inconsistent conclusions or stand in direct contradiction to the promising results. It is imperative that we attempt to understand the reasons that multiple studies of the same marker lead to differing conclusions. A variety of problems have been cited to explain these discrepancies, such as general methodological differences, poor study design, assays that are not standardised or lack reproducibility, and inappropriate or misleading statistical analyses often based on sample sizes too small to draw meaningful conclusions (McGuire, 1991; Fielding *et al*, 1992; Burke and Henson, 1993; Concato *et al*, 1993; Gasparini *et al*, 1993; Simon and Altman, 1994; Gasparini, 1998; Hall and Going, 1999). For example, in retrospective studies, patient populations are often biased towards patients with available tumour specimens. Specimen availability may be related to tumour size and patient outcome (Hoppin *et al*, 2002), and the quantity, quality, and preservation method of the specimen may affect feasibility of conducting certain assays. There can also be biases or large variability inherent in the assay results, depending on the particular assay methods used (Thor *et al*, 1999; Gancberg *et al*, 2000; McShane *et al*, 2000; Paik *et al*, 2002; Roche *et al*, 2002). Statistical problems are commonplace. These problems include underpowered studies or overly optimistic reporting of effect sizes and significance levels due to multiple testing, subset analyses, and cutpoint optimisation (Altman *et al*, 1995).

Unfortunately, many tumour marker studies have not been reported in a rigorous fashion, and published articles often lack sufficient information to allow adequate assessment of the quality of the study or the generalisability of study results. Such reporting deficiencies are increasingly being highlighted by systematic reviews of the published literature on particular markers or cancers (Brundage *et al*, 2002; Mirza *et al*, 2002; Riley *et al*, 2003a, 2003b, 2004; Burton and Altman, 2004; Popat *et al*, 2004).

The development of guidelines for the reporting of tumour marker studies was a major recommendation of the US National Cancer Institute and the European Organisation for Research and Treatment of Cancer (NCI-EORTC) First International Meeting on Cancer Diagnostics (From Discovery to Clinical Practice: Diagnostic Innovation, Implementation, and Evaluation) that was convened in Nyborg, Denmark in July 2000. The purpose of the meeting was to discuss issues, accomplishments, and barriers in the field of cancer diagnostics. Poor study design and analysis, assay variability, and inadequate reporting of studies were identified as some of the major barriers to progress in this field. One of the working groups formed at the Nyborg meeting was charged with addressing statistical issues of poor design and analysis, and reporting of tumour marker prognostic studies. The guidelines presented here are the product of that committee. The Program for the Assessment of Clinical Cancer Tests (PACCT) Strategy Group of the US National Cancer Institute has also strongly endorsed this effort (http://www.cancerdiagnosis.nci.nih.gov/assessment/).

The reporting guidelines proposed in this paper build upon earlier suggestions (Altman and Lyman, 1998; Gion *et al*, 1999; Altman, 2001a, 2001b; Riley *et al*, 2003a) as well as educational publications (McShane and Simon, 2001; Simon, 2001; Biganzoli *et al*, 2003; Schumacher *et al*, 2005). They recommend elements and formats for presentation with the objectives of facilitating evaluation of the appropriateness and quality of study design, methods, analyses, and improving the ability to compare results across studies. Similar to the successful CONSORT initiative for randomised clinical trials (Moher *et al*, 2001), and the STARD statement for studies of diagnostic test accuracy (Bossuyt *et al*, 2003a), these guidelines suggest relevant information that should be provided about the study design, preplanned hypotheses, patient and specimen characteristics, assay methods, and statistical analysis methods. In addition, the guidelines suggest helpful presentations of data and important elements to include in discussions. To be published separately, in an explanatory document, are specific justifications for the need for each of the elements of the recommendations.

We have developed these reporting guidelines primarily for studies evaluating a single tumour marker of interest, often including adjustment for standard clinical prognostic variables. They are largely relevant for studies exploring more than one marker, but they are not intended to specifically address statistical considerations in development of prognostic models from very large numbers of candidate markers. The reason we chose to emphasise prognostic marker studies is that they represent a large proportion of the tumour marker literature and tend to be particularly fraught with problems because they are often conducted on retrospective collections of specimens, and analyses may contain substantial exploratory components. For purposes of this paper, we define prognostic markers to be markers that have an association with some clinical outcome, typically a time-to-event outcome such as overall survival or recurrence-free survival. (Some individuals adhere to a more strict definition of prognostic marker as applying only to the natural history of patients who received no treatment following local therapy.) Prognostic markers may be considered in the clinical management of a patient. For example, they may be used as decision aids in determining whether a patient should receive adjuvant chemotherapy or how aggressive that therapy should be. Predictive markers are generally used to make more specific choices between treatment options. Predictive markers are used as indicators of the likely benefit to a specific patient of a specific treatment. For example, a predictive marker might indicate that a patient expressing the marker will benefit more from a new treatment compared to standard treatment, whereas a patient not expressing the marker will derive little or no benefit from the new treatment. Predictive marker studies usually occur later in the marker development process and there are far fewer published examples. Knowledge of specific treatments received and how those treatment decisions were made become even more critical. In our judgment, the issues in reporting predictive marker studies are complex and different enough from those of prognostic marker studies that we are not willing to claim that these guidelines give predictive marker studies adequate coverage, although we believe that most of the guidance is relevant to such studies too.

The goal of these guidelines is to encourage transparent and complete reporting so that the relevant information will be available to others to help them to judge the usefulness of the data and understand the context in which the conclusions apply. These guidelines are not intended to dictate specific designs or analysis strategies. In general, there is more than one acceptable approach to the design or analysis of a particular study, although these guidelines should help to eliminate some clearly unacceptable options as have been discussed in other papers (Concato *et al*, 1993; Altman *et al*, 1994; Altman and Lyman, 1998; Schumacher *et al*, 2005). For example, unacceptable options include reporting statistical significance of a marker's prognostic effect without acknowledging that the significance testing was preceded by extensive manipulations involving derivation of data-dependent cutpoints or variable selection procedures. High-quality reporting of a study cannot transform a poorly designed or analysed study into a good one, but it can help to identify the poor studies and we believe that it is an important first step in improving the overall quality of tumour marker prognostic studies.

## MATERIALS AND METHODS

Initial ideas for key elements to be addressed in the guidelines were assembled from literature citing empirical evidence of inadequate reporting or problematic analysis methods (Hilsenbeck *et al*, 1992; Altman *et al*, 1994, 1995; Simon and Altman, 1994) based on published reviews of tumour marker studies. Ideas were also generated by reviewing similar reporting guidelines that have been produced for other types of medical research studies (CONSORT, QUOROM, MOOSE, STARD) (Moher *et al*, 1999, 2001; Stroup *et al*, 2000; Bossuyt *et al*, 2003a). Three individuals from the working group (LM, DA, GC) wrote a first draft to serve as a starting point for discussion by the full group. Comments on drafts were made by the full group on a conference call and through multiple e-mail exchanges. A very preliminary draft was presented to the PACCT Strategy Group in January 2001. In response to comments, the guidelines were shortened, reformatted, and recirculated to the full committee. They were posted to the PACCT website (http://www.cancerdiagnosis.nci.nih.gov/assessment/progress/clinical.html) for public comment and circulated to attendees of the NCI-EORTC Second International Meeting on Cancer Diagnostics (Conference on the Development of New Diagnostic Tools for Cancer) that was held in Washington, DC in June 2002. In February 2003, three committee members (DA, LM, WS) met for 2 days to make further revisions. The version produced in that February meeting was sent to the full committee for final comment. The version presented here incorporates those final comments and was approved by the full committee.

## RESULTS

 Table 1 shows the recommendations for reporting studies on tumour markers. Specific items are grouped under headings: Introduction, Materials and Methods, Results, and Discussion, reflecting the relevant sections of a published scientific article. Further details about the recommendations and explanatory material will be provided in a separate article.

As noted in item 12, a diagram may be helpful to indicate numbers of individuals included at different stages of a study. As a minimum, such a diagram could show the number of patients originally in the sample, the number remaining after exclusions, and the numbers incorporated into univariate and multivariable analyses.

## DISCUSSION

The reporting guidelines presented here are the result of a collaborative effort among statisticians, clinicians, and laboratory scientists who are committed to improving and accelerating the process by which tumour markers that provide useful information for management of cancer patients are adopted into clinical practice. In addition to the authors of this paper, we gratefully acknowledge the contributions of many individuals with whom we have had informal discussions regarding these guidelines and who have been supportive of this effort. All of us participating in the development of these guidelines are actively involved in the design, conduct, and analysis of studies involving tumour markers. Collectively, we serve as editors and reviewers for numerous scientific journals that publish tumour marker studies, we serve on programme committees for international meetings, as decision-makers for funding agencies, participants in national and international committees charged with evaluating and prioritising tumour markers for further study or making recommendations for clinical use, and are actively involved in our own research involving tumour markers. As editors, reviewers, and programme and advisory committee members, we have struggled with having to make decisions when insufficient information is provided about study design or analysis methods. As individual investigators, we have experienced the frustration of trying to interpret often confusing literature to guide our own research programmes.

There are consequences of poor study reporting for the research community as a whole. Poorly designed or inappropriately analysed studies can attract undeserved attention when they produce very dramatic, but unfortunately incorrect results. In contrast, some carefully designed and analysed studies have been overlooked because they produced less dramatic, but perhaps more accurate and realistic results. The poor quality of reporting of prognostic marker studies may have contributed to the relative scarcity of markers whose prognostic influence is well-supported. Thorough reporting is required no matter what methods of design and analysis are used. Thorough reporting does not solve problems of poor design or analysis that are being reported; rather, it just fairly describes what problems may exist and need to be considered in interpretation. It is our hope that these guidelines will be embraced and used by journal editors, reviewers, funding agencies, decision-making bodies, and individual investigators.

These guidelines have been labelled as applying to clinical prognostic studies. Not all of the elements apply to studies conducted in earlier phases of marker development (Hammond and Taube, 2002), for example early marker studies seeking to correlate a new marker with other clinical variables or existing prognostic factors. However, our recommendation is that investigators conducting early marker studies should strive to adhere to as many of the reporting guidelines as applicable in their situation, and the guidelines might also suggest issues that will be important for them to consider in planning follow-up studies on their investigational markers. Studies of markers that can be used to predict the success of particular therapies, such as molecular-targeted therapies, need additional considerations. It is our opinion that predictive marker studies should generally be conducted within randomised trials, require a sufficient (usually larger) effective sample size, and assays should be in a more advanced state of development. The CONSORT statement for randomised clinical trials can serve as a starting point for reporting guidelines for predictive marker studies, but additional issues relating to the marker assays must be addressed. It is our feeling that more stringent and specific guidelines need to be developed for reporting studies of predictive markers. Such studies will be considered in somewhat more detail in the planned explanatory paper.

It may not be possible to report every detail for every study. For example, it is often difficult to provide detailed patient inclusion/exclusion criteria or treatment information in retrospective prognostic marker studies using archived tumour specimens. The impact of such missing information must be judged in the specific context of the study and its stated conclusions. For example, a ‘pure’ prognostic study should be conducted in a group of patients who have not received any systemic adjuvant therapy, but treatment information is often missing or unreliable in retrospective studies. In these cases, it is important to recognise that apparent ‘prognostic’ effects may be influenced by potential treatment by marker interactions. The key point is that there must be a clear statement of what is and what is not known. In addition, it was beyond the scope of these guidelines to recommend specific details that should be reported for each of the major classes of marker assays, for example, immunohistochemistry, *in situ* hybridisation methods, or DNA-based assays. There is an ongoing effort to define such assay-specific checklists by another working group evolving from the NCI-EORTC International Meetings on Cancer Diagnostics.

Some of the reviewers suggested that the guidelines should promote full public access to data, possibly even individual-level data. We have chosen not to include this issue in the current scope of the guidelines even though we view movement in this direction as generally positive. One concern is that if a study was poorly designed or inadequately reported, making its data publicly available may simply propagate bad science. Good study design and data quality have to come first. We do recognise the potential benefits of promoting full public access to good quality data. It would allow verification of published analysis methods and results and would facilitate alternative analyses and meta-analyses. Attainment of these goals would be helped significantly if guidelines 10 and 11 were strictly applied, so that statistical analysis methods were described in sufficient detail to allow an individual independent of the original research team to reproduce the results of the study if supplied with the raw data. For extensive analyses, it is possible that some of this information would have to be provided as supplementary material available outside of the main published report, for example, on the journal's or author's website.

While some might view adherence to these guidelines as yet another burden in trying to publish or obtain funding, we would argue that use of these guidelines is more likely to *reduce* burdens on the research community. Making clear what is considered relevant and important to report in journal articles or funding proposals will likely reduce review time, reduce requests for revisions, and help to ensure a fair review process. Furthermore, we consider it as a prerequisite for a thoughtful presentation and interpretation of the results of a specific study and a key aid for a summary assessment of the effect of a marker in a review paper. Most importantly, what greater reduction in burden could there be than to eliminate some of the false leads generated by poorly designed, analysed, or reported studies which send researchers down unproductive paths, wasting years of time and money?

The ultimate usefulness of these guidelines will rely on how widely they are adopted. We are heartened by the enthusiastic responses we received from the several journals who have agreed to simultaneously publish this paper. There is a clear recognition in the community that the time has come (if not long overdue) to improve the quality of tumour marker study reporting and conduct. We hope that many journals will adopt these guidelines as part of their editorial requirements. To the extent that does not happen immediately, we have to rely on authors of journal articles and reviewers of those articles to initiate the movement toward adherence to these guidelines.

We expect that just as tumour marker research will evolve, these guidelines will have to evolve to address new study paradigms and new assay technologies. It is our hope that publication of these guidelines will generate vigorous discussion leading to continually improved versions and ultimately to improved quality of tumour marker studies.

The guidelines presented in this paper are available at http://www.cancerdiagnosis.nci.nih.gov/assessment/progress/clinical.html, as will be other recommendations from the group in due course. As noted, a detailed explanatory paper is in preparation, following the model of similar articles relating to the CONSORT and STARD statements (Altman *et al*, 2001; Bossuyt *et al*, 2003b).

## Figures and Tables

**Table 1:** REporting recommendations for tumour MARKer prognostic studies (REMARK)

**Introduction**
1. State the marker examined, the study objectives, and any prespecified hypotheses.

**Materials and Methods**
**Patients**
2. Describe the characteristics (e.g. disease stage or comorbidities) of the study patients, including their source and inclusion and exclusion criteria.
3. Describe treatments received and how chosen (e.g. randomised or rule-based).

**Specimen characteristics**
4. Describe type of biological material used (including control samples), and methods of preservation and storage.

**Assay methods**
5. Specify the assay method used and provide (or reference) a detailed protocol, including specific reagents or kits used, quality control procedures, reproducibility assessments, quantitation methods, and scoring and reporting protocols. Specify whether and how assays were performed blinded to the study end point.

**Study design**
6. State the method of case selection, including whether prospective or retrospective and whether stratification or matching (e.g. by stage of disease or age) was employed. Specify the time period from which cases were taken, the end of the follow-up period, and the median follow-up time.
7. Precisely define all clinical end points examined.
8. List all candidate variables initially examined or considered for inclusion in models.
9. Give rationale for sample size; if the study was designed to detect a specified effect size, give the target power and effect size.

**Statistical analysis methods**
10. Specify all statistical methods, including details of any variable selection procedures and other model-building issues, how model assumptions were verified, and how missing data were handled.
11. Clarify how marker values were handled in the analyses; if relevant, describe methods used for cutpoint determination.

**Results**
**Data**
12. Describe the flow of patients through the study, including the number of patients included in each stage of the analysis (a diagram may be helpful) and reasons for dropout. Specifically, both overall and for each subgroup extensively examined report the numbers of patients and the number of events.
13. Report distributions of basic demographic characteristics (at least age and sex), standard (disease-specific) prognostic variables, and tumour marker, including numbers of missing values.

**Analysis and presentation**
14. Show the relation of the marker to standard prognostic variables.
15. Present univariate analyses showing the relation between the marker and outcome, with the estimated effect (e.g. hazard ratio and survival probability). Preferably provide similar analyses for all other variables being analysed. For the effect of a tumour marker on a time-to-event outcome, a Kaplan–Meier plot is recommended.
16. For key multivariable analyses, report estimated effects (e.g. hazard ratio) with confidence intervals for the marker and, at least for the final model, all other variables in the model.
17. Among reported results, provide estimated effects with confidence intervals from an analysis in which the marker and standard prognostic variables are included, regardless of their significance.
18. If done, report results of further investigations, such as checking assumptions, sensitivity analyses, internal validation.

**Discussion**
19. Interpret the results in the context of the prespecified hypotheses and other relevant studies; include a discussion of limitations of the study.
20. Discuss implications for future research and clinical value.
